# Motivational interviewing intervention for the management of hypertension: a meta-analysis

**DOI:** 10.3389/fcvm.2024.1457039

**Published:** 2025-01-20

**Authors:** Ju Xu, Xiaoyan Gu, Jiaying Gu, Lijuan Zhao, Mianxian Li, Cheng Hong

**Affiliations:** ^1^ Department of Nursing, Taicang TCM Hospital Affiliated to Nanjing University of Chinese Medicine, Suzhou, China; ^2^ Department of Gastroenterology, Taicang TCM Hospital Affiliated to Nanjing University of Chinese Medicine, Suzhou, China; ^3^ Department of Cardiovascular Medicine, Taicang TCM Hospital Affiliated to Nanjing University of Chinese Medicine, Suzhou, China; ^4^ Department of Cardiovascular Medicine, First Affiliated Hospital of Soochow University, Suzhou, China

**Keywords:** motivational interviewing, hypertension, care, treatment, management

## Abstract

**Background:**

Improving medication compliance and self-efficacy in hypertensive patients is of significant importance for their prognosis. This meta-analysis aimed to assess the role of motivational interviewing in reducing systolic and diastolic blood pressure, self-efficacy, quality of life and medication adherence in hypertensive patients.

**Methods:**

Two authors searched PubMed, Cochrane Library, Clinicaltrials, Embase, Web of Science, China National Knowledge Infrastructure (CNKI), Chinese Biomedical Literature Database, Weipu and Wanfang Database up to May 28, 2024 for randomized controlled trials (RCTs) evaluating the role of motivational interviewing on hypertensive patients. The search was restricted to articles published in English and Chinese languages. RevMan 5.4 software was used for meta-analysis.

**Results:**

A total of 16 RCTs were included. The meta-analysis findings demonstrate that motivational interviewing could reduce both systolic pressure [MD = −3.26, 95% CI (−5.16, −1.36), *P* < 0.001] and diastolic blood pressure [MD = −1.78, 95% CI (−3.48, −0.08), *P* < 0.001] levels in hypertensive patients, while simultaneously enhancing their self-efficacy [MD = 2.92, 95% CI (1.84, 4.00), *P* = 0.001], quality of life [MD = 6.99, 95% CI (3.25, 10.74), *P* = 0.003], and medication compliance [OR = 4.30, 95% CI (1.53, 12.10), *P* = 0.003]. No significant publication bias across the synthesized outcomes were found by Egger regression analyses (all *P* > 0.05).

**Conclusions:**

Motivational interviewing has been shown to effectively reduce blood pressure in the short term among individuals with hypertension, while simultaneously enhancing their self-efficacy, quality of life, and adherence to medication regimens.

## Introduction

Hypertension is one of the most common chronic diseases worldwide and is also one of the major public health issues globally (1). The prevalence of hypertension among people over 18 years old in our country has exceeded 30%, and the population with hypertension exhibits the following characteristics: high incidence, high mortality, high disability rate, low awareness, low control rate, and low medication compliance rate (2–4). It is reported that the medication compliance rate of hypertension patients in China is only 42.5% (5). In recent years, although the awareness, treatment rate, and control rate of hypertension patients have improved, they are still at a relatively low level, which is related to the lack of disease knowledge and weak self-management ability among hypertension patients (6, 7). Studies (8, 9) have shown that unhealthy lifestyle behaviors can increase the incidence of cardiovascular complications in patients. By intervening in patients’ behavior and disease cognition, patients' initiative can be improved, self-management ability can be enhanced, and the quality of life can be improved (10, 11). Therefore, helping individuals establish healthy behavioral patterns is particularly important in hypertension prevention and management.

Motivational Interviewing, a counseling approach developed by William R. Miller (12, 13) and Stephen Rollnick (14), is a scientifically validated method that has been recognized for its efficacy in addressing lifestyle-related issues and disease management. It is regarded as a valuable intervention strategy, leveraging motivation enhancement techniques to foster behavior change (15). Its appeal lies in its accessibility and economic viability, which has endeared it to patients and their kin (16). The robust theoretical framework of motivational interviewing has garnered significant attention from clinical nursing professionals and scholars within the field. Characterized by its client-centered and directive methodology, motivational interviewing is specifically tailored to address and clarify the client's ambivalence during the process of behavioral change. This approach emphasizes the importance of understanding and navigating the complexities of ambivalence, thereby enhancing the effectiveness of the intervention (17). Motivational interviewing is instrumental in fostering the adoption and sustenance of healthy behaviors among hypertensive patients (18). It also aids in the effective management of blood pressure and holds potential for bolstering patients' self-efficacy (19). While the findings are encouraging, the majority of the studies are limited by their small sample sizes. This limitation restricts the generalizability of the conclusions and weakens the evidence supporting the efficacy of motivational interviewing in the management of hypertension. To address this gap, the objective of this study is to evaluate the efficacy of motivational interviewing in reducing systolic and diastolic blood pressure, enhancing self-efficacy, improving quality of life, and promoting medication adherence among patients with hypertension.

## Methods

This meta-analysis was conducted and reported according to the Preferred Reporting Items for Systematic reviews and Meta-Analyses (PRISMA) statement (20). The requirement for ethical approval and written informed consent was deemed unnecessary for this meta-analysis, as it does not involve the collection, analysis, or reporting of individual participant data.

### Literature search strategies

In this study, a comprehensive computer-assisted literature search was performed across multiple databases, including PubMed, Cochrane Library, Clinicaltrials, Embase, Web of Science, China National Knowledge Infrastructure (CNKI), Chinese Biomedical Literature Database, Weipu and Wanfang Database. The search encompassed records from the inception of these databases up to May 28, 2024, employing a strategic combination of Medical Subject Headings (MeSH) and key textual terms. The search strategy for this study was formulated as follows: (“motivational interviewing” OR “motivation interview” OR “MI” OR “motivational counseling”) AND (“hypertension” OR “high blood pressure”).

### Inclusion and exclusion criteria

The inclusion criteria for this meta-analysis were as follows: The inclusion criteria for this meta-analysis, as determined in the past and reorganized according to the PICOS framework, were as follows: Population (P): Adults (age ≥18years old) who had been diagnosed with hypertension, according to the “Guidelines for the Diagnosis and Treatment of Hypertension” established jointly by the World Health Organization (WHO) and the International Society of Hypertension. Hypertension was identified by a systolic blood pressure of ≥140 mmHg and/or a diastolic blood pressure of ≥90 mmHg. Intervention (I): The studies had to involve an intervention group that utilized motivational interviewing as part of the treatment approach. Comparison (C): There had to be a control group receiving standard care, which could include routine medical treatment without the addition of motivational interviewing. Outcomes (O): The studies had to report on outcome measures relevant to the impact of motivational interviewing on hypertensive patients. These outcomes included blood pressure levels, self-efficacy, quality of life, and medication compliance. Study Design (S): Only RCTs that investigated the impact of motivational interviewing on hypertensive patients were considered for inclusion in the meta-analysis.

The exclusion criteria for this meta-analysis were as follows: Review articles and case studies; literature with incomplete data; and duplicate publications.

### Literature screening and data extraction

In the process of literature screening and data extraction, two independent reviewers meticulously assessed the literature in accordance with pre-established inclusion and exclusion criteria, followed by a thorough cross-verification. Any discrepancies were resolved through collaborative deliberation or by seeking the guidance of subject matter experts. Utilizing a custom-designed form, we systematically extracted pertinent data, encompassing the following elements: authorship, year of publication, geographical context, duration of study, nature of interventions, temporal aspects of intervention delivery, frequency of intervention application, identity of intervention facilitators, and the pertinent outcome measures.

### Assessment of literature quality

Two researchers independently utilized the Cochrane Risk of Bias tool (21) to evaluate the studies, with any disagreements resolved through consultation with a third-party expert. The evaluation encompasses several aspects including the generation of random sequences, allocation concealment, implementation of blinding, incomplete reporting of data, selective reporting, and other potential biases. Each component can be rated as having a “low, medium, or high risk of bias”.

### Statistical analysis

In this investigation, we utilized the RevMan 5.4 software to conduct a meta-analysis. The assessment of heterogeneity across the study outcomes was performed through a heterogeneity test. The absence or minimal presence of heterogeneity was inferred when the *P*-value was greater than or equal to 0.1 and the *I*^2^ statistic was below 50%, thereby justifying the application of a fixed-effects model. Conversely, when the *P*-value was less than 0.1 and the *I*^2^ statistic exceeded 50%, substantial heterogeneity was identified, prompting the execution of subgroup analyses or sensitivity analyses to elucidate the underlying causes. In these instances, a random-effects model was employed. For the analysis of continuous variables, the mean difference (MD) was selected as the metric for effect size, while the odds ratio (OR) was utilized for categorical data to quantify the effect size. The results for all effect sizes were reported alongside a 95% confidence interval (CI) to provide a measure of precision and statistical confidence. The threshold for statistical significance in this meta-analysis was set at a *P*-value of less than 0.05.

## Results

**Figure 1:**
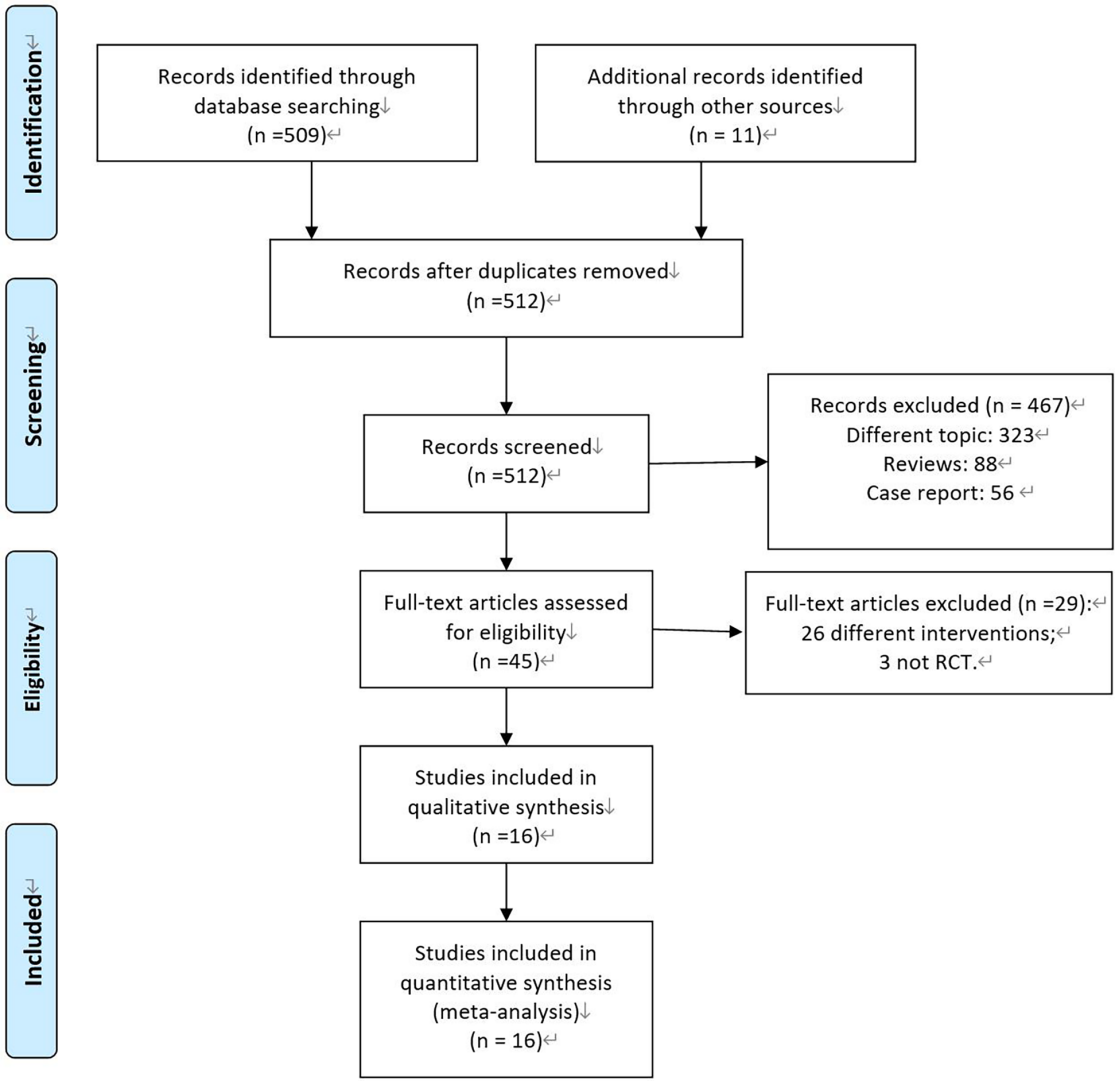
PRISMA flow diagram of RCT selection.

As shown in Figure 1, the study initially retrieved a total of 520 relevant literatures. After excluding duplicate records using EndNote software, 512 remained. After an initial screening based on titles and abstracts, 467 articles were excluded. After reading 45 full texts in detail, 16 RCTs (22–37) were finally included based on the inclusion and exclusion criteria.

### Characteristics of included RCTs

As shown in Table 1, the 16 included studies originated from China, the United States, Ireland, and Denmark. All studies included in the review used motivational interviewing as the intervention method. The duration of the intervention ranged from one month to 18 months, with each session lasting between 10 and 40 min. The most commonly reported study outcomes were the levels of systolic and diastolic blood pressure after the intervention.

**Table 1:** Characteristics of included RCTs.

Study ID	Country	Sample size		Intervention frequency	Intervention duration	Intervention implementer	Outcomes
		Motivational interviewing group	Control group				
Boutin-foster et al., 2016	USA	90	87	Not reported	12 months	Trained research assistants	Blood pressure
Cao et al., 2012	China	47	48	1 time/2 weeks, 20 min/times	6 months	Senior nurses with more than 10 years of service	Blood pressure; quality of life
Hardcastle et al., 2013	UK	203	131	Total 5 times, 20–30 min/times	18 months	Trained researchers and caregivers	Blood pressure
Hedegaard et al., 2015	Denmark	231	285	A total of 3 interviews were conducted in the 1st, 2nd and 7th months, respectively.	12 months	Family nurse	Blood pressure; medication compliance.
Huang et al., 2021	China	135	135	1 time/2 weeks, 30 min/times	6 months	Clinical nurses	Self-management level
Landry et al., 2015	USA	134	135	Once every three months	18 months	Trained researchers and caregivers	Blood pressure
Lei et al., 2014	China	54	53	1 time/2 weeks, 20 min/times	3 months	Medical staff in community health service centers	Blood pressure
Lei et al., 2022	China	60	61	Once a month	6 months	Trained researchers and nurses	Disease cognition, self-management behavior
Liang, 2022	China	43	43	Once a month	6 months	Clinical and community nurses	Blood pressure; self-efficacy
Ma et al., 2014	China	60	60	A total of 8 times, 3,040 min/times	6 months	Clinical nurses	Blood pressure; medication compliance.
Murphy et al., 2009	Ireland	360	405	Individualized intervention plan	18 months	Researchers and nursing staff	Blood pressure
Ogedegbe et al., 2008	USA	95	95	1 time/3 months, 3,040 min/times	12 months	Training and Research Assistant	Blood pressure
Shao et al., 2014	China	40	40	Total 3 times, 20 min/times	6 months	Training and Research Assistant	Blood pressure; quality of life
Zhang et al., 2015	China	38	37	Once per week (1st month), once per month (from 2nd month), 30 min/times	3 months	Medical staff who had received the motivational interviewing training	Self-efficacy; medication compliance;
Zhao, 2013	China	44	42	1–2 months, 3–4 times/month; 3–5 months, 2 times/month; 6 months, 1 time/month	6 months	Trained personnel with professional title of nursing supervisor or above	Blood pressure; medication compliance
Zuo et al., 2017	China	40	40	1 time per week, 3 times in total, 15 min/times	1 month	Professionally trained nursing staff	Medication compliance; quality of life

**Figure 2:**
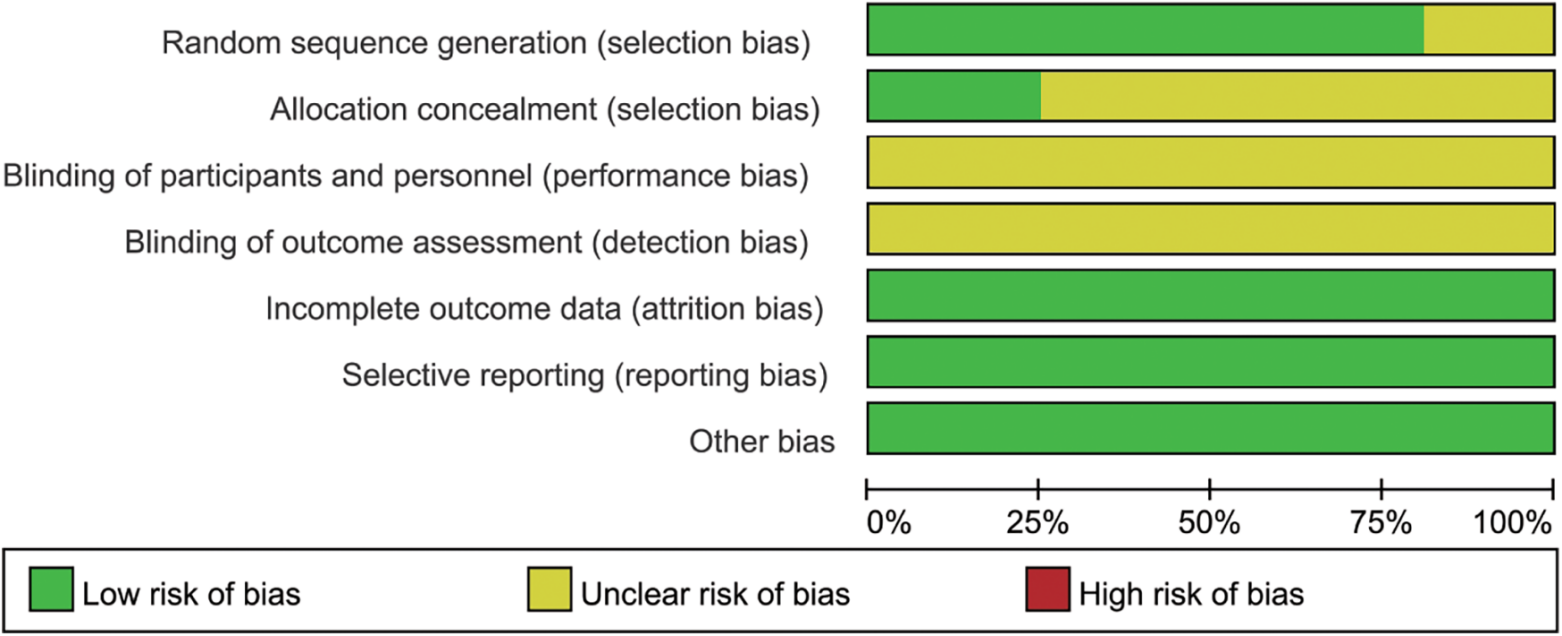
Risk of bias graph.

**Figure 3:**
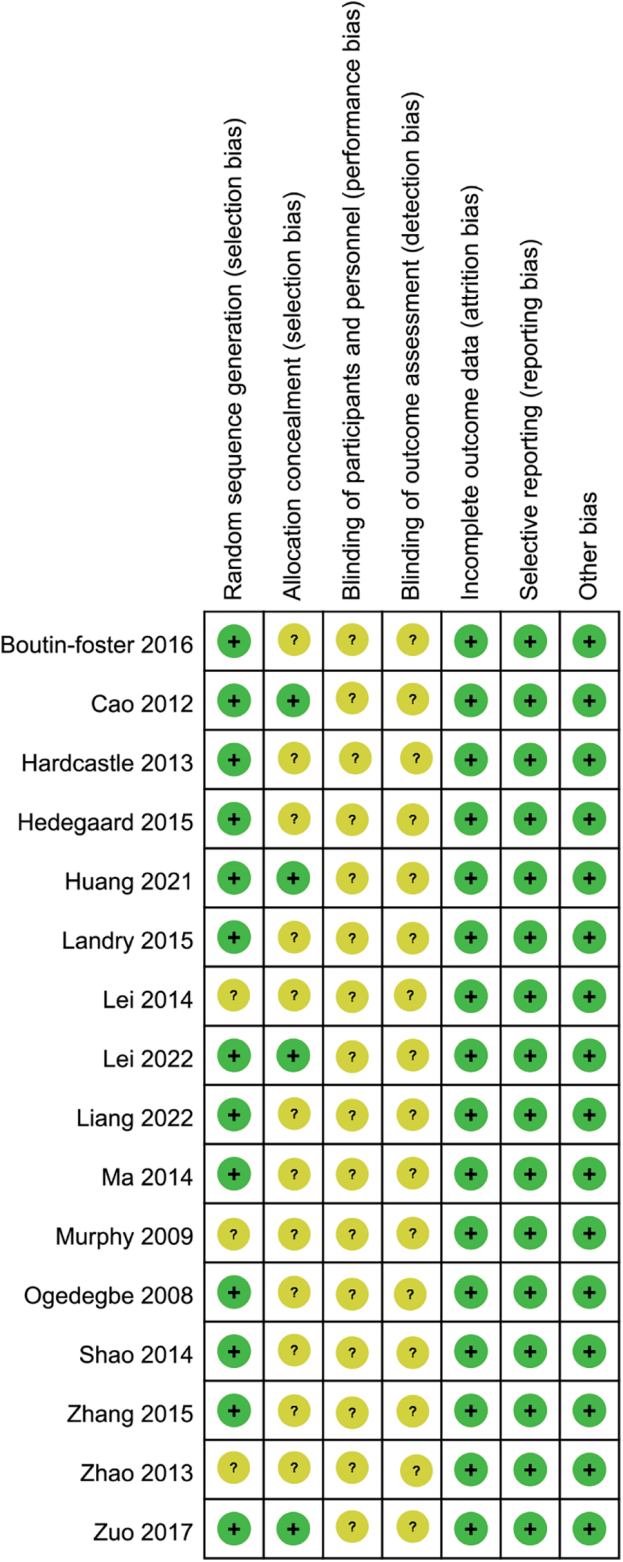
Risk of bias summary.

As depicted in Figures 2, 3, while RCTs discussed in this analysis had explicitly stated the adoption of a random study design, it was noteworthy that three of these studies had failed to provide details regarding the specific techniques employed for generating the randomization sequence. This lack of transparency could have potentially impacted the reliability of the study outcomes. Additionally, four studies had been found to have implemented measures for allocation concealment, a critical component in ensuring the integrity of the randomization process. Given the intrinsic nature of the motivational interviewing intervention, it had been deemed impractical to blind both the participants and the interventionists to the study conditions. This was a common challenge in trials involving psychological interventions, where the interactive nature of the therapy made blinding difficult. Despite this limitation, it was reassuring to observe that all studies included in the analysis had reported comparable baseline data across all groups, which helped to mitigate concerns regarding selection bias. Furthermore, each study had diligently documented patient attrition and dropout rates, which was essential for assessing the overall integrity of the study and the generalizability of its findings. Upon thorough examination, no other potential sources of bias had been identified within the studies reviewed.

**Figure 4:**
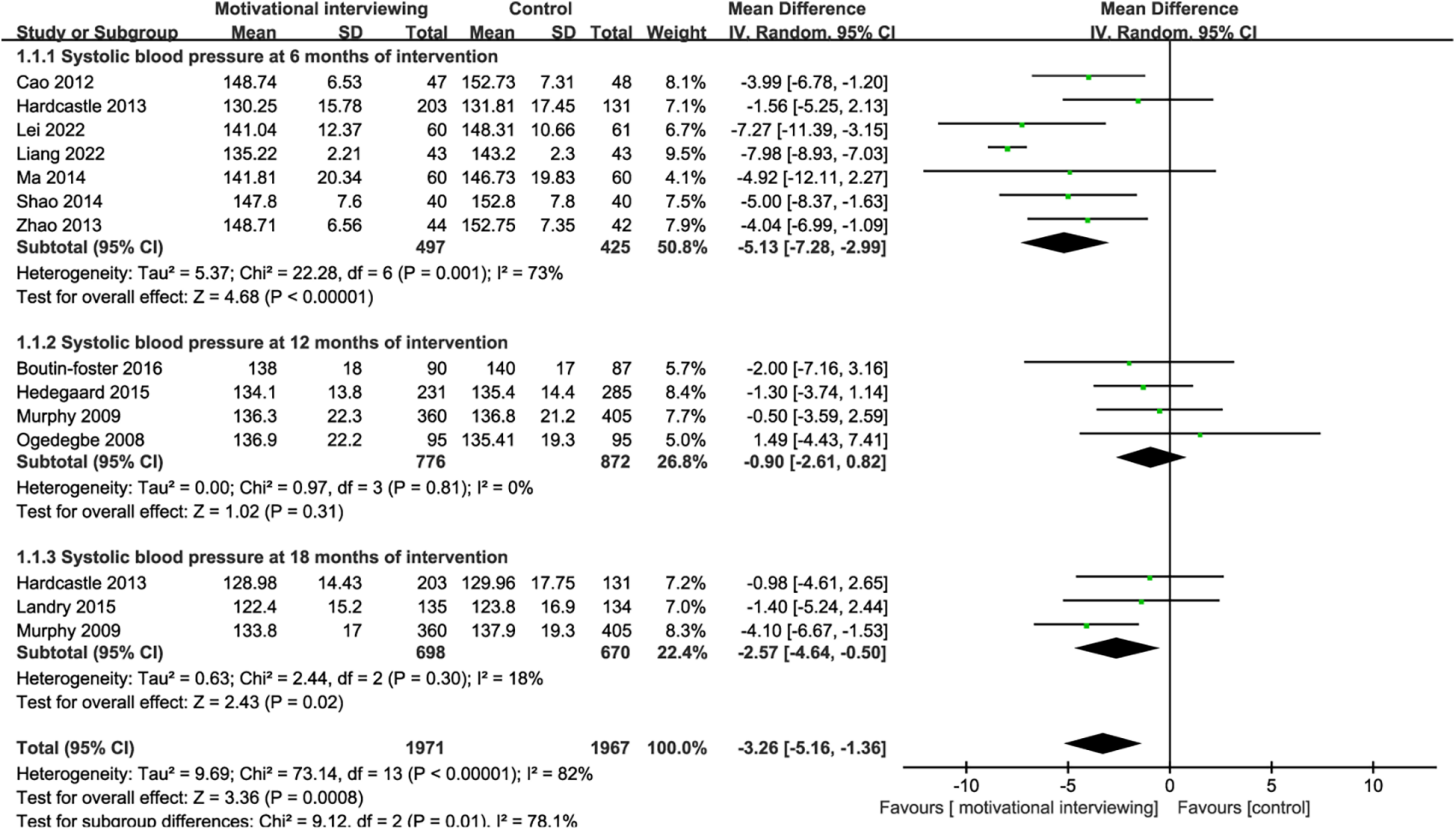
Forest plot for systolic blood pressure.

12 RCTs reported the impact of motivational interviewing on the systolic blood pressure of hypertensive patients. There was significant heterogeneity among the studies (*I*^2^ = 82%, *P* < 0.001), leading to the use of a random-effects model for the meta-analysis. The results indicated that motivational interviewing intervention was effective in reducing the systolic blood pressure of hypertension patients [MD = −3.26, 95% CI (−5.16, −1.36), *P* < 0.001, Figure 4]. Due to the varying intervention durations across the included studies, we conducted subgroup analyses based on the intervention times. Subgroup analysis indicated that motivational interviewing intervention was effective in reducing the systolic blood pressure of hypertension patients at 6 months[MD = −5.13, 95% CI (−7.28, −2.99), *P* < 0.001] and 18 months[MD = −2.57, 95% CI (−4.64, −0.50), *P* = 0.02, Figure 4C] of intervention, no significant effects on the systolic blood pressure at 12 months [MD = −0.90, 95% CI (−2.61, 0.82), *P* = 0.31] were found.

**Figure 5:**
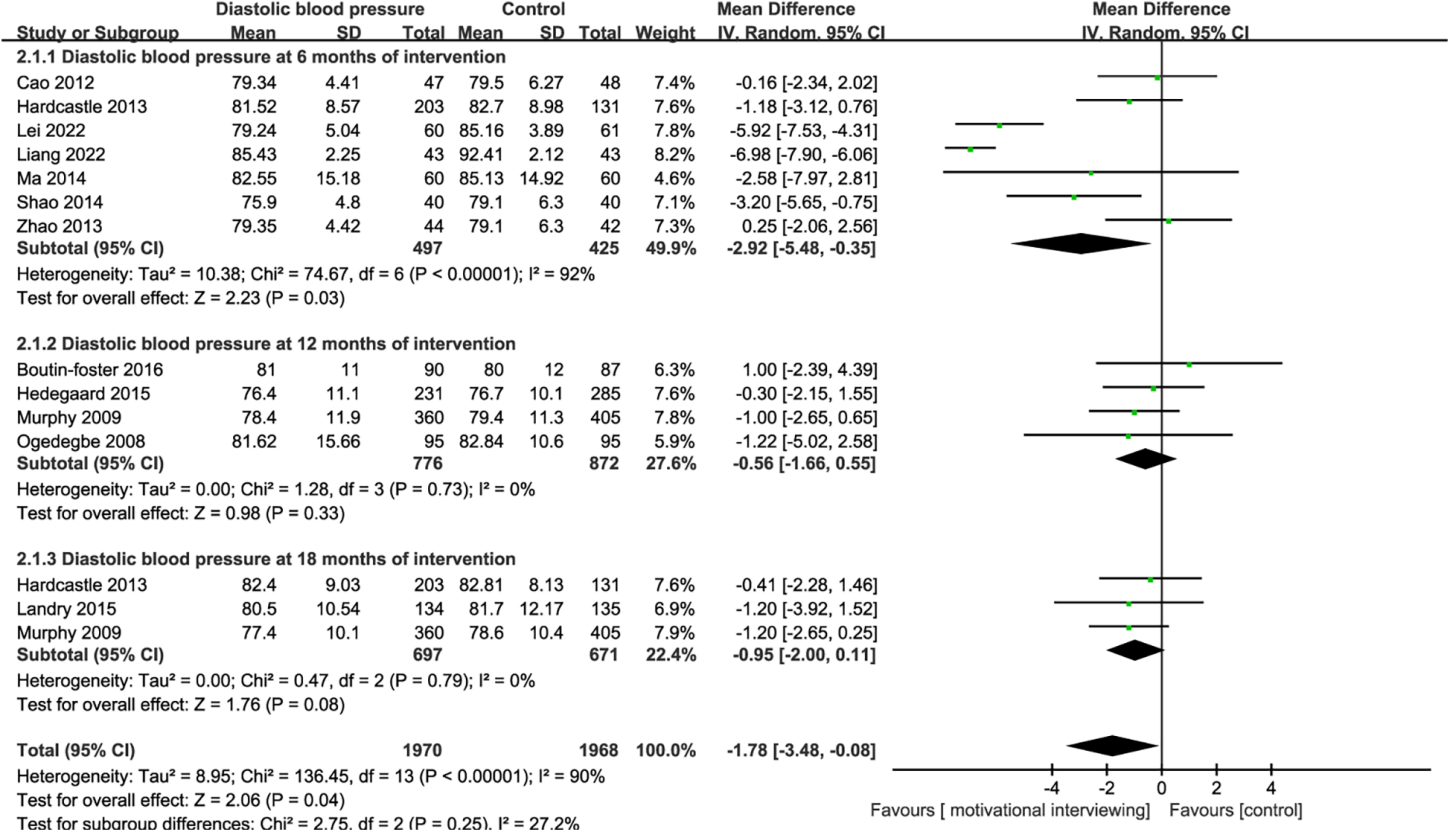
Forest plot for diastolic blood pressure.

10 RCTs reported the impact of motivational interviewing on the diastolic blood pressure of hypertensive patients. There was significant heterogeneity among the studies (*I*^2^ = 90%, *P* < 0.001), leading to the use of a random-effects model for the meta-analysis. The results indicated that motivational interviewing intervention was effective in reducing the diastolic blood pressure of hypertension patients [MD = −1.78, 95% CI (−3.48, −0.08), *P* < 0.001, Figure 5]. Due to the varying intervention durations across the included studies, we conducted subgroup analyses based on the intervention times. Subgroup analysis indicated that motivational interviewing intervention was effective in reducing the diastolic blood pressure of hypertension patients at 6 months[MD = −2.92, 95% CI (−5.48, −0.35), *P* < 0.001] of intervention, no significant effects on the diastolic blood pressure at 12 months[MD = −0.56, 95% CI (−1.66, 0.55), *P* = 0.33, Figure 5B] and 18 months [MD = −0.95, 95% CI (−2.00, 0.11), *P* = 0.08] were found.

**Figure 6:**
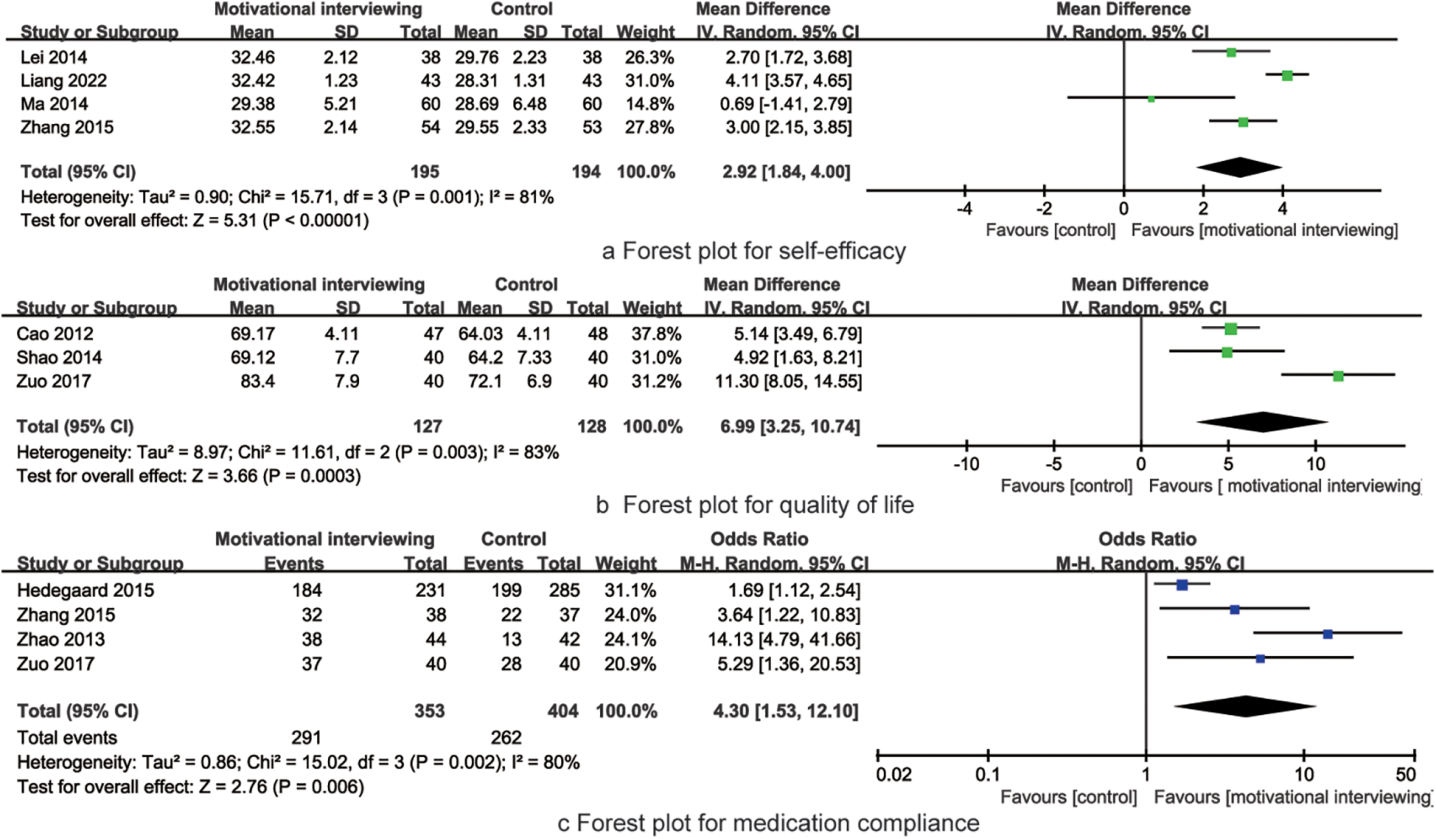
Forest plot for self-efficacy, quality of life and medication compliance.

4 RCTs reported the impact of motivational interviewing on the self-efficacy of hypertensive patients. There was significant heterogeneity among the studies (*I*^2^ = 81%, *P* = 0.001), leading to the use of a random-effects model for the meta-analysis. The results indicated that motivational interviewing intervention was effective in improving the self-efficacy of hypertension patients [MD = 2.92, 95% CI (1.84, 4.00), *P* = 0.001, Figure 6A].

3 RCTs reported the impact of motivational interviewing on the quality of life of hypertensive patients. There was significant heterogeneity among the studies (*I*^2^ = 83%, *P* = 0.003), leading to the use of a random-effects model for the meta-analysis. The results indicated that motivational interviewing intervention was effective in improving the quality of life of hypertension patients [MD = 6.99, 95% CI (3.25, 10.74), *P* = 0.003, Figure 6B].

4 RCTs reported the impact of motivational interviewing on the medication compliance of hypertensive patients. There was significant heterogeneity among the studies (*I*^2^ = 80%, *P* = 0.002), leading to the use of a random-effects model for the meta-analysis. The results indicated that motivational interviewing intervention was effective in improving the medication compliance of hypertension patients [OR = 4.30, 95% CI (1.53, 12.10), *P* = 0.003, Figure 6C].

**Figure 7:**
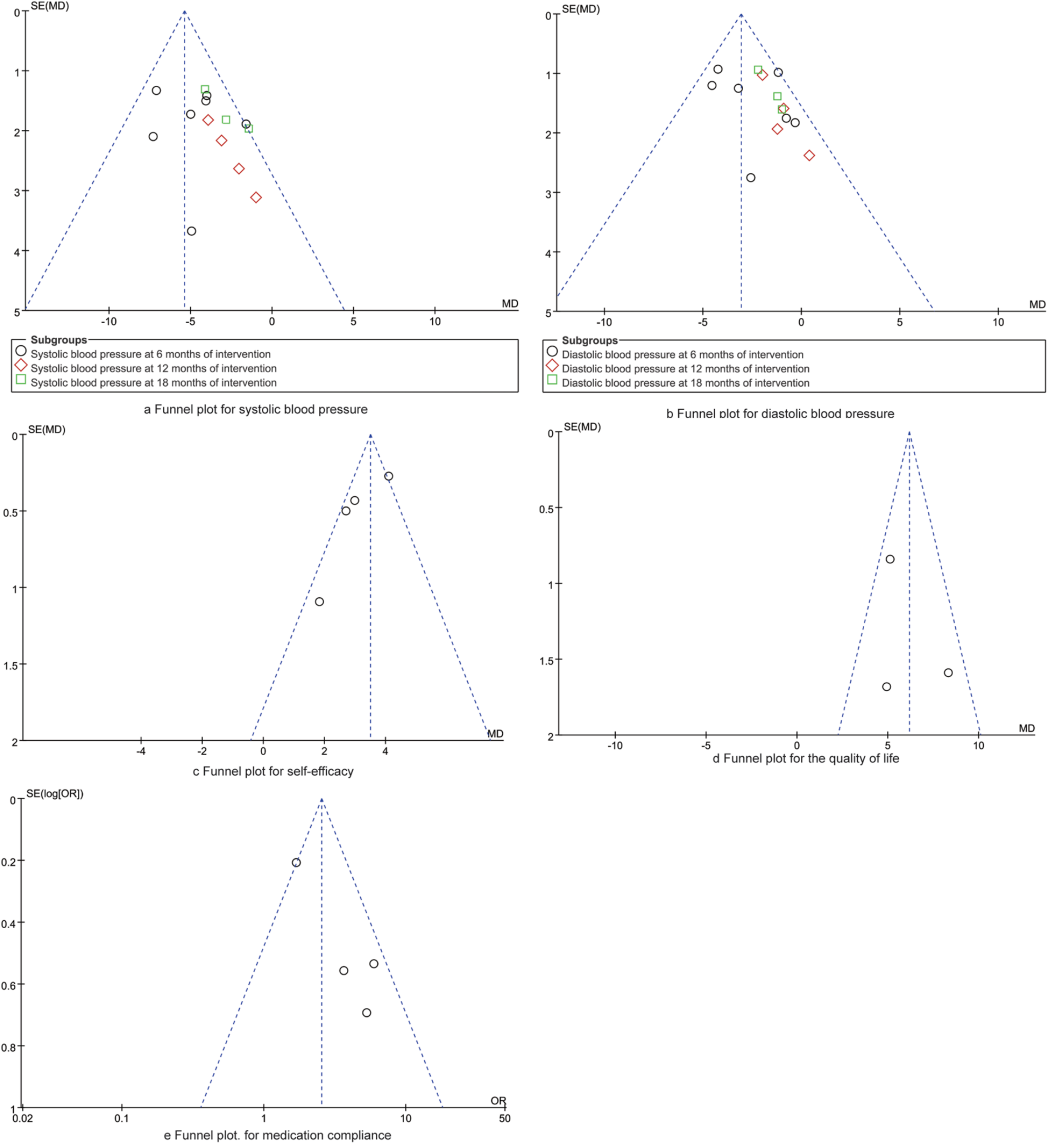
Funnel plots for synthesized outcomes.

As presented in Figure 7, the symmetrical distribution of dots within the funnel plot suggests a balanced and comprehensive representation of the studies included in the analysis. Furthermore, the Egger regression analysis yielded results indicating no significant publication bias across the synthesized outcomes (*P* > 0.05).

## Discussion

Hypertension is a chronic disease that often goes unnoticed until it leads to severe health complications (38, 39). When hypertension symptoms are mild or not noticeable, patients might not realize how serious their condition is and could unintentionally neglect its management. This unawareness can result in poor self-care and self-management, which are key to regulating blood pressure and stopping the disease from getting worse (40, 41). Research (42) has indicated that although basic health education can boost a patient's comprehension of their illness, it doesn't always lead to better self-management practices. This indicates a significant challenge in chronic disease management, such as hypertension, where there's a disconnect between knowing what to do and actually doing it. The meta-analysis indicates that motivational interviewing is effective at reducing blood pressure in patients with hypertension. By boosting patients' self-efficacy—their confidence in managing their condition—this method can lead to better medication adherence, healthier lifestyle decisions, and an enhanced quality of life.

The results of this meta-analysis suggest that motivational interviewing is associated with a reduction in blood pressure levels among patients. However, it is important to note that there were variations in the control of blood pressure at various time points throughout the study. In the early stages of motivational interviewing intervention, patients become aware of the importance of blood pressure control. After being provided with personalized and gradual guidance by the interviewer on issues related to blood pressure control, patients' cognition of hypertension is enhanced, and their intrinsic motivation is stimulated, leading to behavioral changes (43). Patients increasingly recognize the importance of blood pressure monitoring and adopt healthier lifestyle practices, which consequently lead to a significant reduction in their blood pressure levels. Following the commencement of the motivational interviewing intervention, it has been observed that within six months, patients' self-awareness may wane into a state of complacency, leading to intermittent fluctuations in blood pressure control. Nevertheless, as time elapses, with the ongoing support of the motivational interviewing practitioner, patients' behaviors and lifestyle choices evolve into more consistent patterns. This development, coupled with an enhanced sense of self-efficacy, enables them to effectively manage their blood pressure levels over the long term (44). Furthermore, motivational interviewing strengthens supervision and guidance on patients' execution of behavior change plans, affirms their successes, and helps patients address difficulties encountered during the behavior change process, thus playing a motivational and reinforcing role (45).

Previous research (46) suggests that improving self-efficacy is crucial for enhancing patient behavior and health outcomes, with the enhancement of self-efficacy and self-management considered an ideal model for the control of hypertension. Motivational Interviewing has been shown to increase self-efficacy and medication adherence among hypertensive patients (47). The principal factors contributing to suboptimal medication adherence among hypertensive patients include the belief that long-term medication regimens are excessively challenging and a deficiency in self-confidence regarding their capacity to maintain adherence over time (48). Therefore, a strong belief in medication adherence is vital for patients. Motivational interviewing, based on respect for the patient's thoughts and emotions, employs a range of communication skills to encourage patients to explore their inner conflicting emotions in a harmonious atmosphere and therapeutic environment, identifying areas of weak self-efficacy (49). Motivational interviewing tailors its approach to individual differences, instilling confidence in patients to address their own issues; it guides patients in self-encouragement and self-motivation, thereby stimulating their inner potential and enhancing self-efficacy, which in turn strengthens the patient's medication compliance (50). The main factors affecting the quality of life in hypertensive patients are medication adherence, psychological status, sleep quality, self-efficacy, lifestyle, and the level of knowledge about hypertension (51, 52). Upon a multifaceted assessment of these determinants by the motivational interviewing practitioner, a viable and holistic intervention strategy can be delineated. Through systematic guidance, patients are enlightened about the criticality of punctual medication adherence, lifestyle modifications, emotional regulation, and the incorporation of appropriate physical activity for the management of blood pressure. This approach underscores the significance of self-management in disease control, culminating in lowered blood pressure and an enhanced quality of life for the patients (53, 54).

This meta-analysis has several limitations that warrant consideration. Firstly, a limitation of this study is that the protocol was not registered with the PROSPERO database, which could have offered a transparent and publicly accessible record of our research methodology and outcomes. This omission may impact the generalizability of our findings and the feasibility for other researchers to replicate our study. We acknowledge this shortcoming and recommend that future studies should prioritize registration in PROSPERO to enhance the credibility and transparency of their research. Secondly, the current study only searched for literature in Chinese and English, and due to language factors, there may be an incomplete collection of documents. Thirdly, several of the RCTs incorporated in the analysis did not delineate the precise methodology for generating the random sequence and concealing allocation, which introduces an elevated risk of bias into the study's findings. Finally, although this meta-analysis established strict criteria for the inclusion and exclusion of literature, the lack of a standardized motivational interviewing intervention protocol, variations in the training received by researchers in motivational interviewing, and differences in patient education levels, as well as the duration and frequency of motivational interviewing interventions, may be one of the reasons for the clinical heterogeneity observed. In current clinical practice, the frequency and duration of motivational interviewing interventions vary, as does the content of the interventions. The interviewers' skills also show a wide range of proficiency, and these factors may all exert some influence on the study outcomes. Thus, future studies should aim to refine the content of motivational interviewing interventions, determine the optimal frequency and duration of sessions, and develop a standardized system for conducting these interviews. It would also be beneficial for research to incorporate both quantitative and qualitative methods. Timely documentation of patients’ internal feelings during the motivational interviewing process is crucial. Post-intervention, patient interviews should be conducted to gather their subjective experiences.

## Conclusions

In conclusion, the results of this meta-analytic investigation highlight the effectiveness of motivational interviewing in reducing blood pressure levels in the short term (up to six months) among individuals with hypertension. Concurrently, this approach has been shown to enhance patients' self-efficacy, quality of life, and adherence to medication regimens. Given its economic feasibility and practical applicability, motivational interviewing should be considered for integration and adoption within clinical practice. Nevertheless, the limited evidence on the optimal timing and frequency for achieving the best outcomes from motivational interviewing highlights a need for further research. Future studies should focus on refining these methods, establishing standardized interview protocols, and developing thorough training programs for healthcare providers. These efforts are crucial to adapt motivational interviewing to the varying educational levels and disease stages of the hypertensive patients.

## Data Availability

The original contributions presented in the study are included in the article/Supplementary Material, further inquiries can be directed to the corresponding author.
